# Drug Delivery Strategies for Antivirals against Hepatitis B Virus

**DOI:** 10.3390/v10050267

**Published:** 2018-05-17

**Authors:** Latavia Singh, Sunaina Indermun, Mershen Govender, Pradeep Kumar, Lisa C. du Toit, Yahya E. Choonara, Viness Pillay

**Affiliations:** Wits Advanced Drug Delivery Platform Research Unit, Department of Pharmacy and Pharmacology, School of Therapeutic Sciences, Faculty of Health Sciences, University of the Witwatersrand, 7 York Road, Parktown, Johannesburg 2193, South Africa; latavias68@gmail.com (L.S.); sunaina.indermun@wits.ac.za (S.I.); mershen.govender@wits.ac.za (M.G.); pradeep.kumar@wits.ac.za (P.K.); lisa.dutoit@wits.ac.za (L.C.d.T); yahya.choonara@wits.ac.za (Y.E.C.)

**Keywords:** Hepatitis B virus, intracellular drug delivery, liver targeting, hepatocyte, asialoglycoprotein receptor, nanoparticles, cell-penetrating peptides

## Abstract

Chronic hepatitis B virus (HBV) infection poses a significant health challenge due to associated morbidity and mortality from cirrhosis and hepatocellular cancer that eventually results in the breakdown of liver functionality. Nanotechnology has the potential to play a pivotal role in reducing viral load levels and drug-resistant HBV through drug targeting, thus reducing the rate of evolution of the disease. Apart from tissue targeting, intracellular delivery of a wide range of drugs is necessary to exert a therapeutic action in the affected organelles. This review encompasses the strategies and techniques that have been utilized to target the HBV-infected nuclei in liver hepatocytes, with a significant look at the new insights and most recent advances in drug carriers and their role in anti-HBV therapy.

## 1. Introduction

A significant threat to patients suffering from chronic hepatitis B virus (HBV) infection lies in the emergence of cirrhosis, often leading to hepatocellular cancer, portal hypertension, or liver failure. Ten genotypes of HBV have been identified, labeled A through J, with genotypes A, B, and C being most prevalent [1,2]. Anti-HBV therapy, over the last few years, promised an enhanced effectiveness against HBV. Drugs such as interferon-α2B, PEGylated interferon-α2A, lamivudine, adefovir, tenofovir, entecavir, and telbivudine have all been recognized and acknowledged for their treatment and direct antiviral activity against chronic HBV infection [2,3]. The principal aims that need to be addressed to attain a favorable therapeutic approach in an HBV infection should include a reduction in the viral load to undetectable levels, a decrease in the rate of progression of the disease, and a reduced rate of evolution of drug-resistant HBV. The mechanics of targeted drug delivery are foremost in consideration for parenteral administration in addition to shielding therapeutics from degradation and untimely elimination. These mechanics are drug delivery vehicles consisting of soluble carriers such as synthetic polymers, particulate carriers such as micro- and nanoparticles, and target-specific recognition moieties such as monoclonal antibodies.

Classes of drug targeting fall into two groups, namely active and passive targeting. Active targeting utilizes certain interactions at the target site, such as those of ligand–receptor and antigen–antibody binding [4], or in other cases, signals such as temperature or magnetic fields that can be applied externally. Passive targeting involves adjusting carrier systems’ physicochemical properties to that of the physiological and histological features of the target site. At the target site of action, in this case being the liver, a drug is transported by its carrier. For this seemingly straightforward and simple approach to work, multiple fundamental requirements (nonspecific interactions, access to the target site, drug release, and suitability) must be satisfied. This article therefore reviews the currently developed drug delivery strategies for the treatment of chronic HBV infection and provides a broad discussion on their effectiveness to decrease HBV viral counts in the human body. Emphasis is also provided on the drugs currently being utilized, in addition to the novel drug delivery platforms that have been produced with the aim of providing a rationale between the various drugs being utilized in combination with the advanced drug delivery systems employed.

## 2. Site-Specific Liver Targeting

Hepato- or liver targeting does not necessarily reflect hepatocyte targeting, as other cells in the liver such as the Kupffer cells possess the ability to phagocytose. Therefore, carriers need to be modified chemically to achieve the ability of encompassing liver cell type specificity [5]. The asialoglycoprotein receptor (ASGP-R) has been amongst the most distinguished and investigated of all receptors and was first corroborated by Ashwell and Morell [6]. The ASGP-R has provided a location to accommodate cell–cell intercommunication that is membrane-bound. It allows for targeting specificity of various therapeutic agents, and has been the research focus for HBV uptake [7]. Several attempts have been made to label carriers (polymers, human serum albumin, and recombinant high density lipoproteins) with ASGP-R-specific ligands (galactose, lactose, acetylgalactosamine, asialofetuin) to design specific carriers for drug and gene delivery to hepatocytes [8].

Organ-specific targeted drug delivery has been achieved using the linear aminopolysaccharide chitosan and its derivatives. Systems that target the liver employ passive trapping of the drug-loaded delivery device by the reticuloendothelial system (RES) or active targeting established through recognition between hepatic receptors and the ligand-bearing drug-loaded delivery device. In one such study, lactosaminated *N*-succinyl-chitosan was synthesized and its potential as a liver-specific drug carrier assessed [9]. It was found that this carrier accumulated and distributed throughout the liver as a result of interaction with the ASGP-R.

Lactose conjugated to polyion complex micelles consisting of polyethylene glycol grafted to chitosan also indicated promising potential as a liver-specific nanocarrier to deliver the drug diammonium glycyrrhizinate [10]. A study undertaken by Lin and coworkers [11] modified the surface of chitosan nanoparticles by conjugating it with glycyrrhizin. The conjugation occurred through a process of oxidation of the glycyrrhizin with sodium periodate [11]. In vitro studies showed localization of the glycyrrhizin-conjugated chitosan nanoparticles in hepatocytes, and the intracellular uptake amounted to 4.9 times higher than that of hepatic nonparenchymal cells. The nanoparticle dose as well as the incubation time were significant markers of the cellular uptake process. Liver targeting was achieved by a specific interaction between the glycyrrhizin and the hepatocytes: a ligand–receptor interaction.

**Figure 1:**
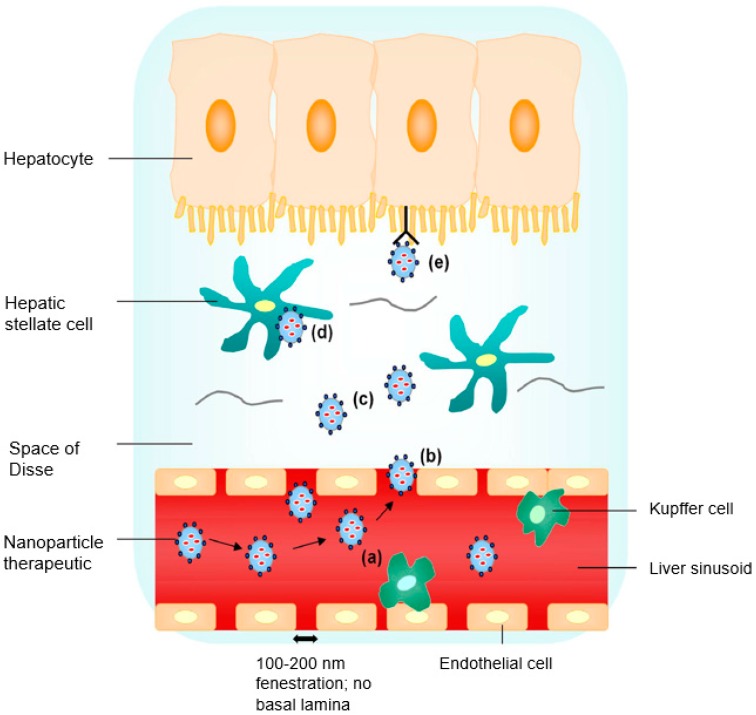
Liver targeting by nanoparticle (NP) therapeutics: (**a**) nanosized particles of less than 200 nm with specific functionalities aid in the evasion of premature Kupffer cell clearance; (**b**) nanosized particles extravasate into the space of Disse through sinusoidal fenestrations in basal lamina absence; (**c**) a high local concentration of NP therapeutics diffusing across the loosely organized extracellular matrix in the space of Disse; (**d**) nonspecific endocytic uptake; and (**e**) receptor-mediated uptake by the hepatocyte (reproduced with permission from [14], © Elsevier B.V. Ltd., 2010).

High-density lipoproteins (HDL) present another effective hepatic targeting carrier. HDL takes up phospholipids and cholesterol from the body’s peripheral tissues and delivers them to the liver hepatocytes via the apolipoprotein (apo) A-l, the major lipoprotein component in HDL. A study undertaken by Biessen and coworkers [12] designed divalent and trivalent cluster glycosides attached to the antiviral nucleoside 9-(2-phosphonylmethoxyethyl) adenine (PMEA). This conjugation promoted ASGP-R affinity [12]. Data from this work indicated that more than 90% of the therapeutic agent was taken up by liver parenchyma cells, thereby reducing its localization in other tissues significantly. Dextran conjugation is also seen in a favorable light. Synergistically, modification of dextran with galactose and mannose results in its selectivity for hepatocytes and Kupffer cells, respectively [5]. Liver-specific treatment and hepatocellular carcinoma (HCC)-targeting of doxorubicin is possible when the polymers used are functionalized with galactosamine [13]. Figure 1 depicts and follows the movement of a therapeutic drug-loaded delivery system to liver hepatocytes.

Various drug carriers often fail in delivering drugs to extravascular sites. A strategic plan would be to use low-molecular-weight prodrugs that have the ability to distribute themselves throughout the body, but are cleaved intracellularly to the active form of the drug by an organ-specific enzyme. HepDirect prodrugs are a series of phosphate and phosphonate prodrugs that pursue a cytochrome P450-catalyzed oxidative cleavage reaction inside the hepatocytes. These prodrugs are cyclic 1,3-propanyl esters that contain a ring substrate which presents sensitivity to oxidative cleavage by the cytochrome P450, with specificity to CYP3A4 [15].

Studies were also performed to test the potential of bile acids to deliver drugs specifically to the liver, since these bile acids are transported across the plasma membrane in the portal domain [16]. The liver-specific drug chlorambucil was used and covalently linked to 7 alpha, 12 alpha, -dihydroxy-3 beta-(omega-aminoalkoxy)-5-beta-cholan-24-oic acid to form chlorambucil-bile acid conjugates, and studies have revealed the success of bile acid molecules in behaving as carriers for drug molecules and their specificity for the liver. Researchers designed a nanoparticle-based model delivery system that simulated nonviral gene delivery particles with respect to their surface properties in an attempt to identify design constraints to aid next-generation gene delivery to the liver [17,18]. Four nanoparticles were formulated from polystyrene beads, namely Gal-50 and Gal-140, which are galactosylated; and MeO-50 and MeO-140, which are methoxy-terminated. The 50 and 140 denotes the mean diameter of the nanoparticles in nanometers. Galactose is incorporated as a targeting ligand, and provision of serum stability is attained with PEGylation of the nanoparticles. Results showed that a slight anionic, galactose-PEGylated nanoparticle should have a size of up to around 50 nm and 140 nm in diameter in order to selectively target hepatocytes and Kupffer cells, respectively. Overall, these studies and findings support the concept that targeted delivery of antivirals to the liver increases the efficacy of these therapeutic agents in the treatment of viral liver infections and reduces their toxicity profiles in other bodily tissues and organs as a result of systemic exposure [5].

## 3. Conventional Anti-HBV Therapies and Drugs

### 3.1. Cytokines and Nucleot(s)ide Analogues

The innate immune system represents the first line defense of a host cell to viral infection [19]. One such example is the IFN-α cytokine, which has displayed supportive findings toward HBV reduction and suppression of HBV viral copying. IFN-α provides for the restitution of T-helper lymphocyte responses as a result of its antiviral activities and immunoregulatory processes, thus it remains the mainstay cytokine presently utilized in the intervention of chronic HBV. Solely, it allows for anti-hepatitis B e-antigen (HBeAg) seroconversion in 25% of chronic HBV disease cases [20,21]. Researchers examined the impression that IFN left on patients with anti-HBeAg positive chronic HBV disease in terms of disease progression by pursuing reexamination after an average of six years [22]. Conclusive results were a 2.5-fold decline in the progression of HBV. Continuous evaluation of its effects was validated in other studies declaring that IFN treatment stimulates an enlarged survival rate, a diminished possible growth of HCC, and consequently, an improvement of liver histology. Future follow-ups showed a sustained clinical subsidence and hepatitis B surface antigen (HBsAG) seroconversion [23,24]. Research has pointed out that lambda-λ has the potential to be useful for therapeutic purposes in the alleviation of chronic HBV or hepatitis C virus (HCV) [25]. IFN-λ is similar to IFN-α and IFN-β in its antiviral behavior to inhibit HBV when it conveys its effects by means of a distinguishable receptor composite in a modified murine hepatocyte cell line. It also lowered HCV replication in Huh7 cells. Data in a study undertaken by Zoulim [26] assessed the antiviral impact of IFN-α2a or IFN-α2b with attached PEG demonstrating close to 30% HbeAg seroconversion and 3–5% HbsAg seroconversion rates, respectively, with HBsAg seen in around 7% of patients after a six-month follow-up. A combination of NUC therapy (entecavir or tenofovir) with recombinant human IL-7 (CYT107) or with both CYT107 and HBV vaccine (GenHevac B^®^, Pasteur, Merieux, Lyon, France) is now being studied [27].

Nucleot(s)ide analogues can be perceived as prodrugs, as their role as inhibitors of polymerases is generated by the phosphorylation process of their nucleoside triphosphates or diphosphates [28]. Even though nucleot(s)ide analogues suppress HBV replication and prevent liver disease progression, they do not affect HBV RNA transcription from the covalently-closed circular DNA molecule of HBV within the infected hepatocytes. They thus have a limited impact on HBV surface antigen serum levels [29]. Lamivudine (LMV) is a cytidine analogue that when compared to IFN-α, does not have a targeted immunoregulatory outcome, but does show the response restoration of T-helper lymphocytes suited to HBV for the initial months of its therapy. The other nucleotide analogues could also feature the same function [30]. LMV is a generally well-endured antiviral and its treatment conduces a fall in HBV DNA level by 3 to 5 log10 copies/mL following 12 months of treatment equated to starting line measurements, an accompanied prompt removal of HBeAg, and standardized/decreased levels of serum alanine aminotransferase (ALT). Prolonged LMV therapy may result in a drop in the risk of developing liver cirrhosis and HCC [31]. Patients in the USA harboring untreated chronic HBV infection exhibited well-disposed results of HBV effects involving chemical processes in the body with reference to its histological characteristics. HBeAg responses were generally kept up after LMV administration [32]; however, it was shown in another examination that HBeAg responses were not significantly kept up after LMV administration was ceased [33]. LMV therapy is suggested in people who do not show any seroconversion. The problem of drug resistance is seen immensely with LMV and its resistance moderates a higher HBV viral load [26].

One of the nucleotide analogues, adefovir dipivoxil (ADV), proved its ability in decreasing the HBV DNA levels in addition to improving serum hepatic enzyme levels. It is also an option for treatment of LMV-resistant patients with cases of chronic HBV infection [34,35]. Patients with chronic HBV infection were randomly assigned to receive specific dosages of ADV every day for the duration of 48 weeks of a study [36]. Results suggested a histologic liver progression, a receded amount of HBV DNA and ALT, and an increased rate of HBeAg seroconversion. A study demonstrated that solid lipid nanoparticles (SLNs) are novel drug delivery systems for ADV for anti-HBV activity [37]. SLNs provide nonbiotoxicity, drug targeting, and sustained drug release as well as harboring the incorporated compound in a protective fashion, and it is also chemically degradable. A fluorescence marker (octadecylamine-fluorescein isothiocyanate) showed the uptake of SLNs by HepG2.2.15 cells. Compared to the free ADV, a significant drop in levels of HBsAg, HBeAg, and HBV DNA levels in vitro was observed. ADV was reviewed in its treatment of chronic HBV, and it was reported that ADV was found to enhance its therapeutic efficiency after three years of its continued treatment [34].

Entecavir (ETV) is another powerful inhibitor of HBV replication and was approved in 2005 for its use against active HBV and liver disease including affirmation of a continuous rise in serum ALT. It also has an amicable side-effect profile and a lesser chance of drug-resistance progression [26,38]. The long-term productiveness of ETV monotherapy in patients with detectable HBV DNA levels was explored by Zoutendijk and coworkers [39]. Conclusive findings by this study suggested that ETV treatment could be extended after 48 weeks, particularly in patients with less HBV DNA, as it precipitates a virological reaction in most of these patients.

Tenofovir disoproxil fumarate (TDF) also has a potent antiviral activity against HBV, but has been investigated more in patients who are HIV-positive and have been coinfected with HBV. Many studies have implied that TDF notably reduced HBV viral load and the probability that it is more effective in therapy compared to ADV [26]. ETV and TDF were reviewed in the clinical setting, where it was shown that ETV produced an average of 86% virologic responses in patients until four years of treatment and TDF produced an average of 82% virologic responses until one year and nine months of treatment. Reports on the tolerability revealed commendable safety profiles and lesser occurrences of drug resistance for both ETV and TDF [40]. According to researchers, five years of treatment with TDF is effective in eliminating HBV, possibly leading to the reversion of liver cirrhosis [36].

In a study comprising of treatment-naïve chronic HBV patients, the efficacy and safety of AL-3778 (then called NVR 3-778) was assessed, given alone and in combination with PEG-IFN for 28 days. Dose-related HBV DNA reductions and early HBeAg reductions were shown with these effects and increased when NVR 3-778 was given with PEG-IFN [27].

The nucleot(s)ide telbivudine is known to be affiliated with the largest HBeAg seroconversion rates, with the additional effect of immunomodulation. This makes it similar in character to PEGylated IFN [41]. Serum HBsAg levels, after 36 months of telbivudine treatment, were evaluated in 162 cases that were positive for HBeAg and displayed decreased HBV DNA serum levels in a persistent manner. The rapid fall in serum HBsAg levels in the first 12 months of telbivudine therapy pinpoints patients who are more inclined to acquiring the compete removal of HBsAg [42]. Table 1 summarizes the impression that cytokines and nucleot(s)ide analogues have on HBV.

### 3.2. Thiazolide Anti-Infectives

Thiazolides have recently displayed promise as antiviral therapeutic agents that may amplify existing or forthcoming treatments against hepatitis. Nitazoxanide (NTZ) is a thiazolide anti-infective that has activity against a range of viruses in cell culture models, anaerobic bacteria, protozoa, and helminths. NTZ antiviral exertion was first stumbled upon when patients with AIDS (coinfected with HBV or HCV) were being treated for cryptosporidial diarrhea, as it was the first thiazolide that was primarily used for the treatment of the protozoan *Cryptosporidium parvum*. In studies of patients with chronic HBV, NTZ administration evoked the seroconversion of HBeAg and HBsAg with the overall elimination of HBV DNA levels. In other studies of patients with chronic HCV, the administration of NTZ together with PEGylated IFN-α2a, with or without the addition of ribavirin, manifested the efficiency and acceptable safety of the thiazolide [43,44,45]. Together with the metabolite of NTZ, tizoxanide, and other thiazolides, reports have indicated the effective inhibition of both HBV and HCV replication in standard antiviral assays. NTZ showed activity against both genotypes 1a and 1b of HCV and the frequent LMV- and ADV-resistant HBV mutants as well as certain HCV mutants. It induced a reduction in the Hep2.2.15 cells producing HBV proteins; however, it did not have an effect on HBV RNA transcription [46]. NTZ is currently undergoing preclinical trials [47]. Researchers performed a QSAR examination on a variety of thiazolides and they assessed their interference on HBV replication and activity [48]. Briefly, the broad-spectrum NTZ presented efficacy against viruses, amongst other things; the novel 2-hydroxybenzoyl-*N*-(5-chlorothiazol-2-yl) amide displayed robust and selective HBV replication hindrance and in comparison to some analogous salicyloylanilides, favorable activity against HBV was shown with numerous 4′- and 5′-substituted thiazolides. It has been suggested that the action of removal with substitution of the nitro group found on NTZ with a group that is not reducible will allow for novel thiazolides to be formed that would sustain a wide spectrum of viral activity only, which would play a valuable role in the reduction of HBV [49].

### 3.3. Small Interfering Ribonucleic Acids

Small interfering ribonucleic acids (siRNAs) with an efficient delivery system can overcome barriers and cause the inhibition of gene expression of specific proteins. The mechanism by which this is achieved is known as RNA interference (RNAi). It is a natural procedure that protects the genome by targeting a particular messenger RNA (mRNA) for degradation, thus inhibiting protein synthesis, and therefore, viral gene transcription, expression, and ultimately replication. There are several notable studies that have been conducted concerning siRNA. Merely to achieve siRNA hepatocyte targeting, in one such study, a vehicle was developed: siRNA Dynamic PolyConjugates [50]. This technology involves a membrane-active polymer that confers its activity only until it reaches the endosome acidic environment and acquires the ability to drop off its siRNA cargo precisely to hepatocytes after a simple intravenous injection. Novel hepatotropic nontoxic lipid-based vector systems were generated to deliver chemically unmodified siRNAs to the liver [51]. Triggered PEGylated siRNA nanoparticles were therefore formulated. Due to the PEG coupling, the resulting pH-sensitive oxime linkage causes the release of nucleic acids from the endosomes. Suppression of markers of HBV replication on account of triggered PEGylated siRNA nanoparticles increase by three-fold relative to controls. Results from another study show that siRNA delivery follows a surface charge- and size-dependent manner [52]. In another study, the aim was to optimally design siRNAs targeting HBV, and this was authenticated via quantitative structure–activity relationship (QSAR) analysis methods [53]. Cocktails of siRNAs could also be problem-solving, as combinations of siRNAs would cleave multiple sites on the mRNA target, making it difficult for restoration thereof [54]. Researchers demonstrated a study that showed the effects of an HBV-specific 21-bp siRNA that is directed to the HBsAg region, a site where three prominent viral mRNAs project over one another, on HBV replication in a cell culture system and a mouse model [55]. Results pointed out the marked inhibition of viral antigens, their transcripts, and DNA, and thus HBV replication as a whole.

A study demonstrated an evaluation of synthetic siRNA combinations targeting various sites of HBV transcripts on its replication and antigen expression in vitro [56]. Results showed that the siRNAs targeting the polymerase and precore region specifically inhibited virus replication and antigen expression in a dose-dependent manner. This was done efficiently compared to the use of single siRNAs at the same final concentration. In addition, no apoptotic change was observed in the cells after the combination siRNA treatment. A specialized delivery system known as a SNALP (stable nucleic-acid-lipid particle) containing chemically modified siRNA in its liposomal form was used [57]. These were administered intravenously into mouse hosts of replicating HBV. HBV DNA reductions specifically lasted for days and weeks with this particular dosing. Taking 3 mg/kg/day intravenous injections three times a day reduced serum HBV DNA by >1.0 log10. A mouse model carrying replicating HBV was injected intravenously with synthetic siRNA/apo A-I/1,2-dioleoyl-3-trimethylammonium-propane complexes [58]. The nanoparticles displayed liver specificity with low doses of less than or equal to 2 mg/kg, still rendering effectiveness in only a single treatment with persistence of the therapeutic effect for eight days. Additionally, administration of these nanoparticles significantly diminished viral protein expression by receptor-mediated endocytosis. Researchers in another study coinjected a compounded *N*-acetylgalactosamine-melittin-like peptide with a siRNA compounded to cholesterol that was directed to coagulation factor 7, and validated the reduction of HBV RNA, DNA, and proteins with a lengthy effective continuity [59]. Data from this study validated the use of RNAi-based therapeutics in the treatment of chronic HBV infections. Another recent study added to the illustration of RNAi decreasing HBV by proclaiming viral clearance of HBV from the liver of transgenic mice by recombinant adenoviruses expressing HBV-directed short hairpin RNAs [60].

ARC-520, designed to reduce the expression and release of new viral particles and the viral protein load by the mechanism of RNAi, although it had showed efficacy in a chimpanzee chronically infected with HBV [61], the clinical trials were discontinued due to safety concerns regarding the toxicity of the delivery vehicle.

### 3.4. Heteroarylpyrimidines

Heteroarylpyrimidines (HAPs) were identified as powerful inhibitors of the HBV capsid maturation step. The interaction site of the core protein–HAP and the exact point in the replication cycle where the HAP allows its principal function is unknown, however the mode of action is the attachment to and degradation of the core protein. Cell-based HBV replication assays portrayed a greater potency of HAPs than LMV in the therapeutic treatment of HBV [5]. Researchers tested the effect that a HAP (methyl 4-(2-chloro-4-fluorophenyl)-6-methyl-2-(pyridin-2-yl)-1,4-dihydropyrimidine-5-carboxylate) had on HBV capsid protein congregation [62]. Results depict the HAP commanding the detachment of HBV capsids and consequently being capable of relaying diversified effects emanating from the incongruous assembling of the HBV capsid proteins. Therefore, actuating and decontrolling the assemblage of a virus could lead to prevailing antiviral-based treatments. A range of HAPs built on a crystallized arrangement of a capsid–HAP conjugate was formulated to attempt to better understand the HBV capsid assembly and replication in HepG2.2.15 cells in vitro [63]. The kinetics of assembly in vitro corresponded sufficiently with the prohibition of HBV in the cell culture. Results also alluded to contention between suitable and unsuitable assembly because of the interrelation of assembly kinetics and virus restriction. BAY 41-4109 is a HAP that was evaluated for its antiviral activity in transgenic rodents carrying HBV at varying dosages (3–30 mg/kg, b.i.d/t.i.d for 28 days). In relation to LMV, BAY 41-4109 was just as competent in lowering HBV DNA in a dose-dependent fashion. Results also suggested lowered HBcAg in excised hepatic sections in disparity with LMV-treated rodents, indicating that BAY 41-4109 bears no resemblance to the mechanism of action of LMV as an antiviral. BAY 41-4109 pharmacokinetics revealed swift absorption and a 30% bioavailability [64]. BAY 41-4109 was also studied for its causative effect on the intracellular EGFP–core fusion proteins into HepG2 cells. It is conclusive that BAY 41-4109 is a powerful inhibitor of HBV, having numerous effects on the arrangement of the HBV capsid to achieve this antiviral result [65]. However, studies by Wang and coworkers [66] reported GLS4 to be more potent than BAY 41-4109 in vitro, demonstrating an EC50 of 12 nM in stably transfected HepG2.2.15 cells. A phase I study is ongoing, but no clinical results have been reported to date [47]. GLS4, a potent inhibitor of the replication of both wild-type and ADV-resistant HBV mutant strains, is currently undergoing a phase I study in China [47]. Isothiafludine (NZ-4), a leucamide A derivative, has been investigated for the inhibition of HBV replication in HepG2.2.15 cells. NZ-4 was mechanistically shown to increase replication of deficient capsids, devoid of pgRNA [67]. Table 2 reflects the efficiency of thiazolides, siRNAs, and HAPs as HBV eradicators.

### 3.5. Sulfamoyl Benzamide Capsid Assembly Modulators

A relatively new class of HBV nucleocapsid assembly modulators is the sulfamoyl benzamides (SBAs). When compared with BAY 41-4109 and AT-61, SBAs were found to inhibit viral replication in a manner similar to that of the phenylpropenamide derivatives, though the interaction of SBAs with core proteins or capsids is still to be extensively reported on [67]. A study by Ohtsuki and coworkers [69] demonstrated a reduction of HBV DNA similar to that of entecavir when a humanized uPA/SCID mouse model received a monotherapy of NVR 3-778 for six weeks. A synergistic effect was also observed when NVR 3-778 was dosed in combination with PEG-interferon, reducing HBV DNA levels to below the limit of quantitation. Recently, sulfamoyl benzamide capsid assembly modulators in the treatment of HBV have been proven efficacious in Phase IB studies, validating NVR 3-778 as a class of anti-HBV compounds [67].

## 4. Novel Drug Delivery Strategies for Anti-HBV Therapeutics

### 4.1. Nanoparticle Systems

#### 4.1.1. Inorganic Nanoparticles

A study concerned with imaging in liver cancer placed emphasis on a proteoglycan known as glypican-3 (GPC-3), which is involved with enabling cell growth and is found to be overexpressed in HCC. Superparamagnetic iron oxide novel multifunctional nanoparticles were developed, where particles were conjugated to streptavidin and Alexa Fluor 647. It was found via confocal fluorescence microscopy (CFM) that biotin-conjugated GPC-3 monoclonal antibody was confined only to the cellular surface of HepG2 cells expressing GPC-3. The GPC-3 nanoparticle system proved that it can be utilized in imaging for HCC visualization as well as having capability to be used as a vehicle for delivery of therapeutics targeting tumors [70]. Researchers also used magnetic nanoparticle surfaces in another study to prepare multifunctional HCC-targeting agents by blending bis-*N*-hydroxysuccinimide ester and OSu-activated fluorescent dye Cy3. A monoantennary and triantennary galactosyl ligands were each fixed onto the fluorescent magnetic nanoparticles and their uptake into HepG2 and HeLa cells were evaluated by CFM. Results show that this system is a good ligand transporter and that its multivalent ligand assembly improves on the cell interaction with HepG2 receptors. The galactosyl Cy3 magnetic nanoparticles were also found to be noncytotoxic [8]. SiO_2_ nanoparticles were also used for attachment to HBV-like particles in order to transit immune response-regulating agents for targeted treatment [71].

#### 4.1.2. Polymeric Nanoparticles

Poly(vinylbenzyl-*O*-β-d-galactopyranosyl-d-gluconamide) (PVLA) is known to be site-specifically taken up into hepatocytes via ASGPR. Therefore, researchers synthesized a copolymer, poly(*N*-*p*-vinylbenzyl-[*O*-β-d-galactopyranosyl-(1→4)-d-gluconamide]-*co*-*N*-*p*-vinylbenzyl-6-[2-(4-dimethylamino)benzaldehydehydrazono]nicotinate) (P(VLA-*co*-VNI)), and this was tagged with (99m)Tc for liver imaging. Results revealed promising potential for more of its application in the evaluation of liver cell function [72]. Researchers have also achieved site specificity to hepatocytes by synthesizing a galactosylated PEG-graft-PEI derivative via the modification of a biscarbamate cross-linked PEI byproduct with PEG and lactobionic acid. The complex held a galactose moiety for hepatocyte targeting. It could also efficiently incorporate plasmid DNA into nanoparticles. Data displayed its successful targeting to the hepatocytes [73]. In a different study, nanoprecipitation and solvent evaporation techniques were used to prepare cationic poly(lactide) (PLA)-based nanoparticles together with PEI and chitosan as surface coating components. mPEG-PLA-PEI nanoparticles showed the best ability to impede HBV surface antigen and thus allow for transfection of siRNA [52]. Researchers used a double-emulsification technique to prepare HBsAg passively adsorbed onto the surface of cationic PLGA nanoparticles for site-specific delivery of IFN-α to hepatocytes. HbsAg-coated (99m)Tc-labeled PLGA nanoparticles results presented notable liver recovery, in contrast to normal PLGA nanoparticles [74]. The particle size and hydrophobicity effects of porous PLA and PLGA nanoparticles were assessed on cell-mediated and mucosal immune responses. The nanoparticles accommodated a set amount of HBsAg, and administration occurred via pulmonary delivery. Data revealed that hydrophobic particles that were larger in size, greater than 500 nm, derived a potent increase in secretory IgA, IFN-γ, and interleukin-2 levels, as opposed to hydrophilic particles that were smaller than 500 nm. The larger sized hydrophobic particles also showed that they could be more easily taken up into rat alveolar macrophages. The study demonstrated the competence of the nanoparticles to induce augmented immune responses [75].

**Figure 2:**
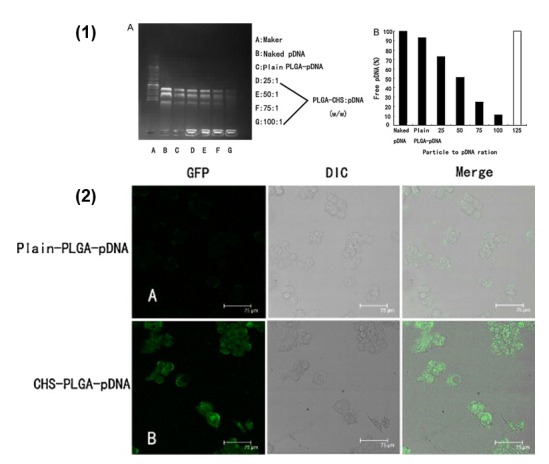
(**1**) PLGA–CHS nanoparticle binding efficiency and loading capacity to adsorb pDNA. (**A**) PLGA–CHS–pDNA complexes with increasing amounts of PLGA–CHS NS were prepared and analyzed for pDNA immobilization ability. Electrophoresis was carried out using 1% agarose gel in TAE buffer containing 0.5 μg/mL ethidium bromide at pH 8. (**B**) The amounts of free DNA were related to naked pDNA (100% mobile) run on the same gel. To quantify the pDNA-immobilization ability, the PLGA–CHS NS/pDNA ratios (*w*/*w*) required for 100% immobilization are compared in this graph (solid bars = percentage of free DNA; white bars = 100% immobilization). (**2**) Confocal laser microscopic images of HepG2.2.15 cells following 48 h transfection with (**A**) plain-PLGA–pDNA NS and (**B**) CHS–PLGA–pDNA NS. Scale bar = 75 μm (reproduced with permission from [76], © Elsevier B.V. Ltd. 2011).

Researchers have also developed polymeric nanoparticles formulated for HBV gene silencing making use of the common biodegradable polymer PLGA again, but in this study, the cationic polymer chitosan is embodied into its matrix. The idea is to advance plasmid DNA loading efficiency and cellular internalization. Conclusive data by Zeng and coworkers [76] revealed better HBV silencing with the chitosan–PLGA system compared to plain plasmid DNA (pDNA) alone or simple PLGA nanoparticles (Figure 2). Furthermore, the chitosan–PLGA system showed a lack of adverse effects.

Micelles were developed in one study for incorporating the lamivudine (LMV) prodrug LMV stearate. LMV stearate was synthesized to increase LMV lipophilicity, due to it being a very hydrophilic drug. Stearic acid-graft-chitosan oligosaccharide micelles were prepared and loaded with LMV stearate. Results showed that the micelles had constrained effects on HBV antigen expression and DNA replication, and this was more noticeable when compared with LMV or its prodrug alone [77]. Acyclovir was also complexed to chitosan-*g*-stearate through the use of a succinate linker for anti-HBV activity. The acyclovir–chitosan-*g*-stearate was able to self-assemble in aqueous solution, constructing micelles. According to data, there was a significant escalation in its inhibitory effect of HbsAg compared to plain acyclovir alone. An all-round success of cellular internalization and anti-HBV effects was observed with the complexation of acyclovir to chitosan-*g*-stearate [78]. Authors also revealed an assuring hepatic-targeted siRNA delivery system for gene expression silencing formulated from *N*-acetylgalactosamine-functionalized mixed micellar nanoparticles. The *N*-acetylgalactosamine micellar nanoparticles were self-aggregated in aqueous solution from *N*-acetylgalactosamine-functionalized PEG-*b*-poly(ε-caprolactone) and cationic poly(ε-caprolactone)-*b*-poly(2-aminoethyl ethylene phosphate) (PCL-*b*-PPEEA). The targeting effect to the hepatocytes was displayed by noteworthy enhanced fluorescent siRNA found in primary hepatocytes, implying positive anti-HBV therapy in liver disease [73]. A drug carrier was synthesized from glycyrrhetinic acid-modified sulfated chitosan, with glycyrrhetinic acid being the targeting ligand to HepG2 cells. The micelles formed from this complex revealed swift and eminent ability for in vivo liver targeting. Moreover, the formed micelles showed specificity for liver cancer cells, in contrast to normal liver cells [79].

### 4.2. Lipids

#### 4.2.1. Ionizable Lipid Nanoparticles

Researchers of a study found that apolipoprotein E, amongst its other roles, also had the ability to pose as an endogenous targeting ligand for ionizable lipid nanoparticles, excluding cationic lipid nanoparticles, with a second means of targeting via an exogenous ligand formed from an *N*-acetylgalactosamine cluster. Both target-specific systems seemed profoundly acceptable in carrying ionizable lipid nanoparticles to the hepatic environment [80].

#### 4.2.2. Cationic Lipids

Novel cationic lipids were synthesized from *N*-cholesteryloxycarbonyl-3,7-diazanonane-1,9-diamine (CDAN) that is conjugated to a dialkylglycylamide moiety to form *N*′,*N*′-dioctadecyl-*N*-4,8-diaza-10-aminodecanoylglycine amide (DODAG). This was used to form lipoplex nanoparticles containing siRNA, and these efficiently led to the in vivo delivery of siRNAs to the liver of transgenic mice, mediating the suppression of HBV replication which was highly comparable to LMV, with minimal observable liver toxicity [81]. In other studies, a cationic lipid–DNA complex (CLDC) was assessed for its capacity to aid HBsAg in extracting immune responses and to cause a decline in HBV DNA levels in transgenic mice. It was triumphant in accomplishing both of these functions [82]. Researchers evaluated the use of a synthetic HBV preS-derived lipopeptide, HBVpreS/2-48(myr) (HBVP), known to be equipped with a coercive ability for liver tropism in site-specificity for liver cell delivery. The lipopeptide was conjugated to PEGylated liposomes (HBVP-Lip). Data pointed out the capability of HBVP-Lip to deliver cargo to hepatocytes with definitive target specificity both in vitro and in vivo [83].

#### 4.2.3. Liposomes

Cationic liposomes have been considered as novel adjuvant systems because they themselves are not adequate as immunostimulating agents. Therefore, ligands that do acquire this function are coalesced with the liposomes, acknowledging them as adjuvants to the functional division of the complex and thus bringing about potential application for enhanced HBV vaccine delivery [84]. In one study, asialofetuin was affixed to cationic liposomes for hepatocyte selectivity, and conjoined to it were diverse cyclodextrins and plasmid DNA for gene transfer. From the cyclodextrins, γ-cyclodextrin complexed with the DNA–asialofetuin liposome showed the most dominant transfection efficiency with zero cytotoxicity, the highest entrapment ratio of the DNA, and the ability to stabilize the membrane of the asialofetuin liposome. γ-cyclodextrin was noted as an amplifier of gene transfer efficiency in liposomes appended with asialofetuin [85]. In a more recent study, Uhl and coworkers [86] delivered Myrcludex B orally using glycerylcaldityltetraether (GCTE) lipids. Promising results were shown in Wistar rats, where an enhanced liver uptake was shown (approximately 7%, 3 h after oral administration).

#### 4.2.4. Lipoplexes

**Figure 3:**
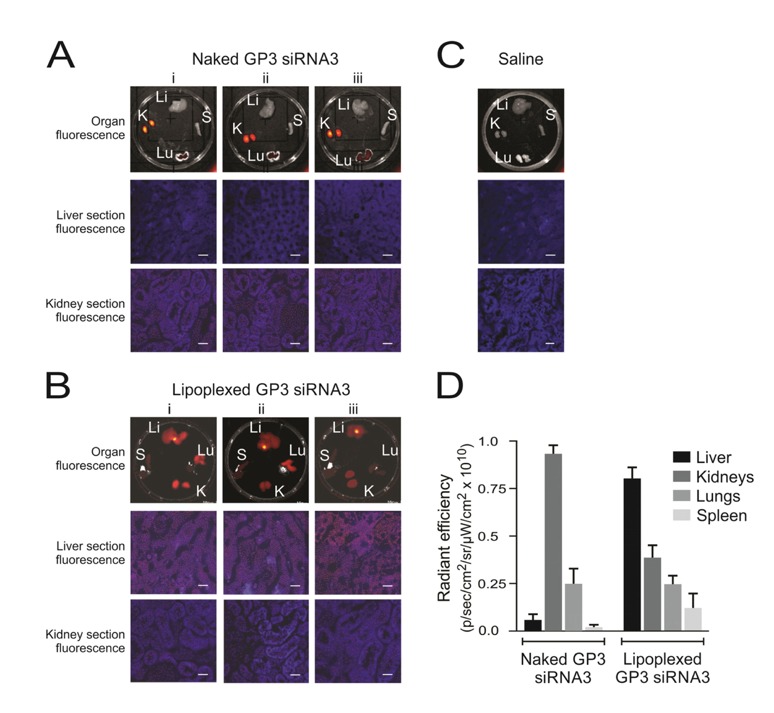
Biodistribution of siRNAs in HBV transgenic mice. Representative fluorescence images obtained from samples harvested 10 min after injection of lipoplexes containing Alexa Fluor 750-labeled siRNAs. Three mice (i–iii) received intravenous injection of the uncomplexed naked labeled siRNA (**A**), and three mice (i–iii) received labeled siRNA within polyglutamate-containing lipoplexes (**B**). One mouse, which received a saline injection, served as the negative control (**C**). Fluorescence detectable in livers (Li), lungs (Lu), kidneys (Ki), and spleens (S) are shown in the top row of images. Microscopy of frozen sections from liver and kidney samples are also shown below. The scale bar indicates 50 μm. Quantitation of fluorescence, radiant efficiency, was measured in the organs of mice given naked guanidinopropyl (GP3)-siRNA3 or lipoplexed GP3-siRNA3 (**D**). Data are represented as the mean radiant efficiency (±SEM) for each organ from the three animals (reproduced with permission from [88], © 2015 Elsevier B.V.).

Researchers have also evaluated the practicality of hindering HBV replication in vivo utilizing the recently reported altitrol-containing class of synthetic siRNAs. They were executed as lipoplexes and assessed in vivo using an HBV transgenic mouse model. Observations and findings revealed success in the silencing of HBV replication, with no toxicity. Results correlated with conceding the future application of altitrol-containing siRNA therapeutic lipoplexes [87]. A study by Marimani and coworkers [88] demonstrated the use of hepatotropic lipoplexes containing siRNAs with guanidinopropyl (GP3) modifications. siRNA biodistribution was assessed by intravenous administration of Alexa Fluor 750-labelled gene silencer lipoplexes. Fluorescence was detected in the kidneys, liver, lungs, and spleen postmortem. Fluorescence in the liver and spleen was barely detectable, with most of the naked labeled GP3 siRNA3 accumulated in the kidneys and lungs. When complexed to the liposomes, hepatic delivery was favored, and diminished fluorescence was detectable in the kidneys, lungs, and spleen (Figure 3). Following systemic intravenous injection into HBV transgenic mice, evidence of toxicity was not observed with appreciable inhibition of viral replication markers.

#### 4.2.5. High-Density Lipoprotein

A study undertaken by Vickers and coworkers [89] illustrated the capability of HDL to transport microRNAs (miRNA). miRNAs, being a novel group of biomarkers, warranted an efficient targeting potential, therefore its export to HDL was achieved by sphingomyelinase. Additional findings revealed that the delivery by means of HDL was reliant on scavenger receptor class B type I. Healthy patients and patients with high cholesterol levels exhibited dissimilar HDL-miRNA effects, and this inspection specified the HDL means of cell–cell intercommunication allowing for miRNA transfer [89]. In other studies, contrast agents for MR imaging in the liver were developed using reconstituted HDL enclosing gadolinium (Gd)-labeled cholesterol as nanoparticles.

This would be advantageous to determine anatomical variations in the liver. The contrast agent nanoparticles bound to HDL receptors on the HepG2 cells would reveal uptake. Researchers also investigated whether the popular drug doxorubicin hydrochloride could be efficiently encompassed into reconstituted HDL for liver targeting. A doxorubicin–HDL compound was formed, and assessments showed that the compound was successfully taken up into liver cells laden with the HDL-specific scavenger receptor class B type 1, had heightened and effective cytotoxicity results against many cell lines, and had the ability to lessen cancer progression to a greater degree than the drug in liposomes. In this case, the reconstituted HDL was effective yet again in drug deliverance targeting the diseased liver [90,91].

#### 4.2.6. Solid Lipid Nanoparticles

Cationic and mannosylated solid lipid nanoparticles (SLNs) were prepared to show potential as a vehicle for HBV vaccine delivery via the subcutaneous route. The mannosylated SLNs displayed superior cellular internalization and a reduced amount of cytotoxicity as well as causing a larger TH1 immune response type [92]. Cationic SLNs were also reconstructed from native low-density lipoproteins and were formed to have application in the target-specific systemic delivery of connective tissue growth factor siRNA (siCTGF). The system was developed for the liver fibrosis treatment in HBV. Fluorescence imaging and single-photon emission computed tomography (SPECT) allowed for biodistribution studies which demonstrated the specific targeting, delivery, and buildup of cationic solid lipid nanoparticles/siCTGF nanocomplexes in the liver [93].

### 4.3. Cell-Penetrating Peptides

**Figure 4:**
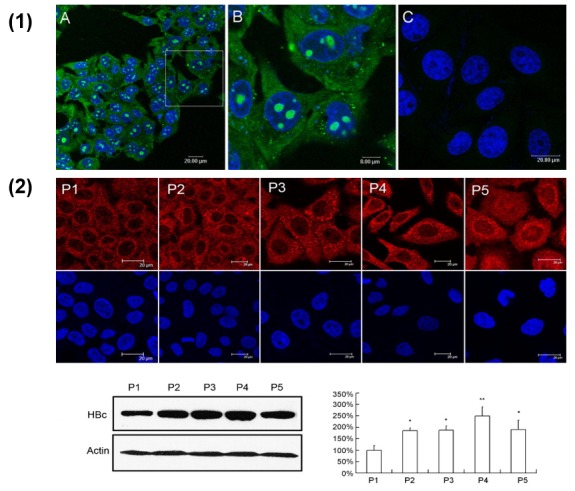
(**1**) Evaluation of cell-penetrating activity of the peptides in the HepG2.2.15 cells by confocal microscope detection. (**A**) HepG2.2.15 cells were treated with 10 μM of FITC-R7-GSLLGRMKGA peptide for 30 min. Nuclei were stained with DAPI (blue); (**B**) shows an enlargement of the white-frame area in (**A**); in (**C**), the HepG2.2.15 cells were treated by 10 μM of FITC-GSLLGRMKGA control peptide without R7. (**2**) Analysis of HBV core protein in HepG2.2.15 cells treated with peptides. Cells were treated with 10 μM of peptides for three days and stained by immunofluorescent staining (upper). Cells were scanned by confocal microscope for subcellular distribution of core protein (red). The core protein was also quantified by Western blot analysis (lower). Optical densities of the core protein were analyzed using QuantityOne software. All values are means ± SD of results from three independent experiments. Values significantly different from control peptide group are indicated by a Student’s *t*-test. * *p* < 0.05; ** *p* < 0.01 (reproduced with permission from [95]).

A study undertaken by Xun and coworkers [94] took advantage of the complimentary execution of cytoplasmic transduction peptide (CTP) in delivering its payloads to hepatocytes. This was carried out by researchers purifying an anti-HBV core single-chain variable fragment welded to CTP, followed by assessment of its potential in HBV inhibition. It markedly reduced HBV DNA levels [94]. Authors developed a series of artificial recombinant peptides, along with the cell-penetrating sequence R7 and various nucleocapsid binding subunits (NBS). Epsilon-aminocaproic acid residue (Acp) was used to link R7 and NBS. The intracellular distribution of FITC-labeled peptide demonstrated that the cell-penetrating peptides were highly efficiently introduced into HepG2.2.15 cells [95]. Conclusive data showed that the synthetic recombinant CPPs holding NBS can enter into cells readily, cause obstruction of the nucleocapsid assembly, and arrest HBV release (Figure 4).

Another novel drug delivery platform with its groundwork on a CPP motif called X-Pep, derived from the extreme *N*-terminal region of the X-protein of HBV, is said to be applicable in having drugs delivered straight to cells specifically [96]. Researchers in another study also coinjected an *N*-acetylgalactosamine-melittin-like peptide (NAG-MLP) with a siRNA compounded to cholesterol that is directed to coagulation factor 7, and validated the oppression of HBV RNA, DNA, and proteins with a lengthy effective continuity. The indication of nearly complete reduction of the 2.1 kb RNA suggests that siRNA was functionally delivered to all cells in which transcription of viral mRNAs was occurring. Thus, data proposed by this system of RNAi-based therapeutics shows its strong potential as a novel therapeutic for chronic HBV infections [59].

### 4.4. Inhibitors of HBV Attachment

In order to prevent HBsAg loss or seroconversion in some patients, research on HBV treatment has focused on the HBV entry process: antibodies that neutralize HBV via interaction with viral surface proteins, inhibitors of viral attachment, and molecules that antagonize NTCP receptor function [97]. The lipopeptide Myrcludex-B, the GMP version of a synthetic lipopeptide derived from the preS1 domain of the HBV envelope protein, has been studied to prevent the spread of HBV spreading postinfection in humanized HBV-infected uPA/SCID mice [98]. Results supported the ability of Myrcludex-B to inhibit the spread of HBV from infected human hepatocytes in vivo as well as to suppress the amplification of the cccDNA pool in initially infected hepatocytes. Other HBV entry inhibitors include: antibodies (HBIG, Ma18/7, KR127, and 17.1.41/19.79.5); attachment inhibitors (heparin, suramin, and SALP); NTCP inhibitors (cyclosporin A, SCYX1454139, and ezetimibe); and bile salts (taurocholic acid). Thus far, HBIG has been approved for the treatment of HBV [97].

## 5. Treatment Endpoint for Chronic HBV Infection

Significant clinical outcomes for the treatment of chronic HBV infection generally takes decades to occur, which makes the prevention of clinical complications a therapeutic priority in HBV-infected patients. ALT normalization and HBV DNA suppression during clinical trials have been determined to be valid surrogate endpoints for assessing the benefits of HBV treatment. However, the durability of these markers is low and therefore they cannot be used as indicators to stop treatment [99]. In HBeAg-positive patients, HBeAg seroconversion (loss of HBeAg and detection of anti-HBe) is considered a valid clinical endpoint, and can be used as an indicator for stopping NUC treatment together with undetectable HBV DNA, in addition to consolidation therapy being completed. For HBeAg-negative patients, HBsAg loss is an ideal endpoint, however the low rate at which this occurs makes it a difficult goal to achieve [99,100]. The development of effective treatment options is therefore a priority to ensure that a greater number of patient reach HBV clinical treatment endpoints. The potential for further research to determine additional endpoints as well as sustained treatment responses also exists to ensure greater clinical outcomes during and after HBV treatment regiments.

## 6. Concluding Remarks

Although the treatment of HBV has seen numerous developments, the development of novel liver-specific drug delivery strategies that prevents the high morbidity and mortality associated with HBV is still in its early stages. There should be a demand for carriers that reach the height of optimization with intracellular targeting, protection of its contents, and efficient delivery thereof. Adequate information has been sought out with respect to receptors that dwell on the specific liver cell types; therefore, with this knowledge and more, receptor-specific ligands can be embodied in the design of nanocarrier systems to achieve heightened efficiency of therapy. A desired characteristic of nanocarriers and an added advantage to anti-HBV therapy is the capacity to incorporate agents together with the required drug for liver imaging. The discovery of novel drug candidates together with their relevant carriers should also prove to be promising in HBV eradication, or at least in significant reduction. However, it should be expected for future anti-HBV therapies to embrace a medley of agents, such as the nucleot(s)ide analogs together with immunostimulants and curative vaccines. Once these ideal goals have been reached and achieved, quality of life will be greatly improved for patients suffering from this debilitating disease.

## Figures and Tables

**Table 1:** Summary of anti-hepatitis B virus (HBV) cytokine and nucleot(s)ide analogue therapeutic agents as a treatment plan.

Therapeutic Agent	Effectiveness as Anti-HBV Therapy	Reference
IFN-α/-PEG/-λ	Suppressed HBV viral copying, restored T-helper lymphocyte responses/immunoregulatory, allows for anti-HbeAg seroconversion, prolonged decline in the progression of HBV, prolonged diminished growth of HCC, sustained clinical subsidence and HBsAG seroconversion, subdued HCV replication	[20,21,22,23,24]
LMV	T-helper lymphocyte response restored, HBV DNA level decline, rapid HBeAg removal, decreased levels of serum ALT, minimal risk of developing liver cirrhosis and HCC, improved liver histology, sustained HBeAg responses after treatment	[26,30,31,32,33]
ADV	Decreased HBV DNA levels, improved serum liver histology, treatment of LMV resistance, decreased ALT, increased HBeAg seroconversion, decreased HBsAg and HBeAg levels, prolonged therapeutic efficacy	[34,35,36,37]
ETV	Potent HBV inhibition, minimal drug-resistance progression, effective long-term, precipitates virological responses, favorable side-effect profile	[26,38,39]
TDF	Potent HBV inhibition, decreased HBV viral load, more effective therapy compared to ADV, sustained virologic responses, favorable safety profile, prolonged liver cirrhosis reversal	[26,36,40]
Telbivudine	Highest HBeAg seroconversion rate, immunomodulation, rapid decrease in HBsAG levels, possible complete removal of HBsAg	[41,42]

IFN-α/-PEG/-λ: interferon-α/PEGylated-interferon/lambda-interferon; anti-HbeAg: anti-hepatitis B e-antigen; HCC: hepatocellular carcinoma; LMV: lamivudine; ADV: adefovir dipivoxil; HBsAG: hepatitis B surface antigen; HCV: hepatitis C virus; LMV: lamivudine; ALT: alanine aminotransferase; ADV: adefovir dipivoxil; ETV: entecavir; TDF: tenofovir disoproxil fumarate.

**Table 2:** Summary of anti-HBV thiazolide, siRNA, and HAP therapeutic agents as a treatment plan.

Therapeutic Agent	Effectiveness as Anti-HBV Therapy	Reference
Thiazolides	Potent and selective HBV replication inhibition, HBeAg and HBsAg seroconversion, decreased HBV DNA levels, treatment in LMV- and ADV-resistance, decreased Hep2.2.15 cell HBV proteins, no HBV RNA transcription effect	[43,44,45,46,48,49]
siRNAs	Gene expression inhibited, viral antigen inhibition, HBV transcript inhibition, decreased serum HBV DNA and RNA levels, suppressed HBV replication markers, mRNA cleavage, polymerase and precore region targeting, effective continuance of treatment	[50,51,54,60]
HAPs	Potent HBV capsid maturing inhibition, HBV core protein degradation, increased potency compared to LMV, potent antigen inhibition effect, misassembly of HBV capsid proteins, dose-dependent decrease in HBV DNA levels, decreased HBcAg	[5,62,63,64,65,68]

SiRNAs: small interfering ribonucleic acids; RNA: ribonucleic acid; mRNA: messenger RNA; HAPs: heteroarylpyrimidines; HBcAg: hepatitis B core antigen.
